# The interplay between STDP rules and anticipated synchronization in the organization of neuronal networks

**DOI:** 10.1186/1471-2202-14-S1-P71

**Published:** 2013-07-08

**Authors:** Fernanda S Matias, Pedro V Carelli, Claudio R Mirasso, Mauro Copelli

**Affiliations:** 1 Departmento de Física, Universidade Federal de Pernambuco, Recife, Pernambuco 50670-901, Brazil; 2 Instituto de Física Interdisciplinar y Sistemas Complejos, CSIC-UIB, Campus Univesitat de les Illes Balears E-07122 Palma de Mallorca, Spain

## 

Neuronal networks are able to synchronize in phase-locking regimes. Depending on the structural connectivity it is possible to determine the sign of the phase difference between oscillatory nodes. Specially in unidirectionally coupled motifs the relative phase difference is usually positive. However it has been shown that in the presence of dynamical inhibitory loops, unidirectionally coupled neuron models may present both positive or negative phase difference [1]. The unusual regime in which the phase difference is negative is called anticipated synchronization (AS) [2]. It means that two neurons or two neuronal populations coupled in a master-slave configuration can oscillate in such a way that the slave leads the master.

These results are important in light of the growing experimental evidence that the synaptic strength between neurons can undergo spike-timing-dependent plasticity (STDP) [3]. In the delayed synchronization regime the master (pre-synaptic) neuron fires a spike before the slave (post-synaptic) neuron, which under STDP rules would facilitate long term potentiation (LTP), whereas in the AS regime the slave neuron fires a spike before the master neuron, contributing to long term depression (LTD) [4]. Since it has been shown previously that a simple 3-neuron motif can undergo a continuous transition from positive to negative values of relative phase via changes in synaptic conductances [1], the interplay between these regimes and STDP mechanisms is likely to play a very significant role in the organization of the system dynamics.

Considering that in certain situations the relative potentiation in strong synapses is less intense than in weak synapses, but the depression do not show this dependence [5], we compare the effects of additive, multiplicative and hybrid rules in our motifs. Together with AS regimes in neuronal populations, STDP rules give synaptic weight distributions that are comparable to experiments in cortex [6,7].
